# Meta-Analysis of the Effects of Yeast Cell Wall Extract Supple-Mentation during Mycotoxin Challenges on the Performance of Laying Hens

**DOI:** 10.3390/toxins16040171

**Published:** 2024-03-30

**Authors:** Alexandra C. Weaver, Daniel M. Weaver, Nicholas Adams, Alexandros Yiannikouris

**Affiliations:** 1 Alltech Inc., 3031 Catnip Hill Road, Nicholasville, KY 40356, USA; ayiannikouris@alltech.com; 2 Independent Researcher, Orrington, ME 04474, USA; dmw1121@gmail.com; 3 Alltech UK, Ryhall Road, Stamford, Lincs PE9 1TZ, UK; nadams@alltech.com

**Keywords:** egg production, hen, mycotoxins, meta-analysis, poultry, yeast cell wall

## Abstract

A random-effects meta-analysis was conducted to investigate the effect of mycotoxins (MT) without or with the inclusion of yeast cell wall extract (YCWE, Mycosorb^®^, Alltech, Inc., Nicholasville, KY, USA) on laying hen performance. A total of 25 trials were collected from a literature search, and data were extracted from 8 of these that met inclusion criteria, for a total of 12 treatments and 1774 birds. Laying hens fed MT had lower (*p* < 0.05) body weight (BW) by −50 g, egg production by −6.3 percentage points, and egg weight by −1.95 g than control fed hens (CTRL). Inclusion of YCWE during the mycotoxin challenges (YCWE + MT) resulted in numerically greater (*p* = 0.441) BW by 12.5 g, while egg production and egg weight were significantly (*p* < 0.0001) higher by 4.2 percentage points and 1.37 g, respectively. Furthermore, economic assessment calculations indicated that YCWE may not only support hen performance but also resulted in a positive return on investment. In conclusion, mycotoxins can play a role in negatively impacting laying hen performance and profitability. Inclusion of YCWE in feed with mycotoxin challenges provided benefits to egg production and egg weight and may support profitability. As such, the inclusion of YCWE could play an important role in minimizing mycotoxin effects and in turn aid farm efficiency and profitability.

## 1. Introduction

Feed quality is an important component of animal production. However, the toxic secondary metabolites produced by some species of molds, known as mycotoxins, can be present and negatively influence the performance and health of birds even with rigorous quality control and monitoring systems in place [1,2,3]. The occurrence of mycotoxins in feed commodities has been well described, with reports indicating contamination frequency of detectable mycotoxins is at 60–80% worldwide [4,5,6]. Along with this high occurrence rate, multiple mycotoxins are likely to be found together which could contribute to an overall increase of the risk associated with those contaminants. As such, it is likely that birds are exposed to mycotoxins in their feed on a daily basis which could hinder optimal performance.

There are many factors that influence mold and mycotoxin development. Pre-harvest, extreme weather patterns, type of farming system, soil management, crop rotation, plant species or hybrid, and pest control can all play a role in the level of mycotoxin challenge [7,8]. Post harvest, high moisture content (>14% moisture) could potentiate mycotoxin challenges in stored ingredients [7,8]. Despite the frequent presence of mycotoxins, a variety of methods exist for minimizing mycotoxin challenges pre-harvest, post-harvest, during storage or processing, and at the animal level [1,9,10].

One of the most practical ways to directly protect animals from mycotoxins is with specific feed additives that work to minimize the harmful effects of mycotoxins through binding or modification [11]. When incorporated into the diet, mycotoxin management feed additives may result in less absorption of mycotoxins from the gastrointestinal tract into the blood and organ systems. There are different categories of mitigation products, including yeast cell wall-based products which have a broad scope for multiple mycotoxin interaction [1,3,11,12]. Specifically, the yeast cell wall extract (YCWE; Mycosorb^®^, Alltech, Inc., KY, USA) that is rich in insoluble parietal carbohydrates comprised mainly of glucans, mannans, and chitin, has shown ability to adsorb multiple mycotoxins in vitro, ex vivo and in vivo [3,11,13,14,15].

In order to better understand the effects of the YCWE mycotoxin mitigation strategy on the performance of laying hens, a meta-analysis was conducted on historical data. Meta-analytical studies provide a way of summarizing results across a body of research to provide a single quantifiable result [16]. As such, this meta-analysis aims to provide an overall assessment on the benefits of using YCWE as part of a mycotoxin management strategy. The objectives of this meta-analysis were to (1) assess the impact of feeding laying hens mycotoxin-contaminated diets (MT) on body weight (BW), feed intake (FI), egg production, and egg weight in comparison to control (CTRL) diets with mycotoxins below the limit of detection in naturally contaminated feed or not added to diets utilizing a pure or culture mycotoxin challenge; (2) to quantify the performance differences with YCWE inclusion during mycotoxin challenges (YCWE + MT) versus MT; and (3) to determine the extent of YCWE use during a mycotoxin challenge to return the performance of laying hens back to CTRL. Additionally, results from this meta-analysis were used to evaluate the possible roles of mycotoxins and YCWE on the economic impact of egg production. To the researchers’ knowledge, this is the first time a meta-analysis study has been conducted with laying hens which evaluates the influence of not only mycotoxins alone but also the use of a mycotoxin mitigation strategy on key performance parameters. 

## 2. Results

### 2.1. Research Characteristics

Following the literature search, 25 trials were obtained that assessed the use of YCWE alongside an MT challenge with laying or breeding hens. Several trials, however, did not report the required performance and production parameters or mycotoxin challenge information as described above, resulting in a total of 12 treatments from 8 trials selected for this meta-analysis. Of this total, 7 were published in scientific journals or scientific conferences as well as a masters thesis (Table 1). One additional available unpublished technical report was included in this meta-analysis to provide a broader range of data in hopes of reducing publication bias [17]. The studies included in the meta-analysis were published over a 14-year period (1999 to 2013) and were conducted in 6 countries (two each from Canada and Italy, and one each from Brazil, India, Serbia, and the United States). There were 1774 hens including 194 fed CTRL; 790 fed MT alone; and 790 fed YCWE + MT. Trials were conducted over 4 to 12 weeks. The average dose rate of YCWE was 1.58 kg/t, with 42% of treatments using 1.0 kg/t and 58% using 2.0 kg/t.

Mycotoxins reported to contaminate the MT rations (Table 1) included aflatoxins (AF; 4 treatments, 0.005 to 3.0 mg/kg), ochratoxin A (OTA; 2 treatments, 0.19 to 0.20 μg/kg), deoxynivalenol (DON; 2 treatments, 2.53 to 12.1 mg/kg), 15-acetyl-deoxynivalenol (1 treatment; 0.5 mg/kg), deoxynivalenol-3-glucoside (1 treatment, 0.3 mg/kg), T-2 toxin (T2; 3 treatments, 0.5 to 2000 mg/kg), fumonisins (FUM; 2 treatments, 0.171 to 25 mg/kg), and zearalenone (ZEA; 4 treatments, 0.33 to 10.36 mg/kg). Trials used diets naturally contaminated with mycotoxins or mycotoxins formed by culture following mold inoculation; however two studies included pure mycotoxins and one did not specify a source. 

### 2.2. Body Weight

**Figure 1:**
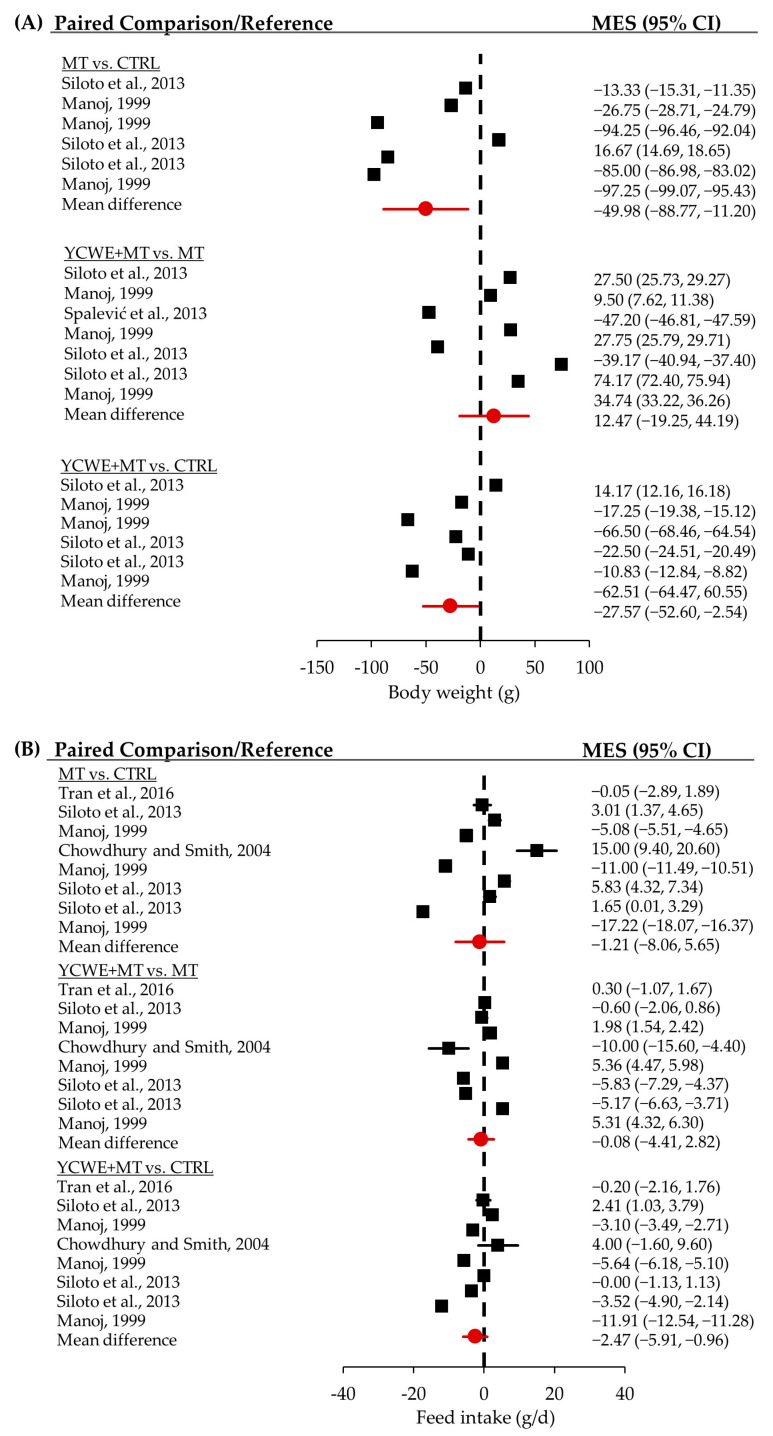
Forest plots from the random-effects meta-analysis [18,19,20,21,24] of the mean differences in (**A**) body weight and (**B**) feed intakefor laying hens fed control without detectable mycotoxins (CTRL), mycotoxins alone (MT), or yeast cell wall extract during mycotoxin challenges (YCWE + MT, Mycosorb^®^, Alltech, Inc.). The mean effect sizes (MES) with 95% confidence intervals (95% CI) between treatments are shown for individual studies (in black) and the overall difference (in red).

Analysis for mycotoxins and YCWE effects on hen BW showed that birds fed MT had significantly lower (*p* < 0.05) BW than those fed CTRL by a mean difference of −50.0 g (Table 2, Figure 1). There was no difference between hens fed YCWE + MT and those fed MT. The BW of hens fed YCWE + MT was lower (*p* < 0.05) than CTRL by −25.6 g. 

### 2.3. Feed Intake

There was no statistical difference between treatment comparisons (Table 2, Figure 1) for FI. 

### 2.4. Egg Production 

**Figure 2:**
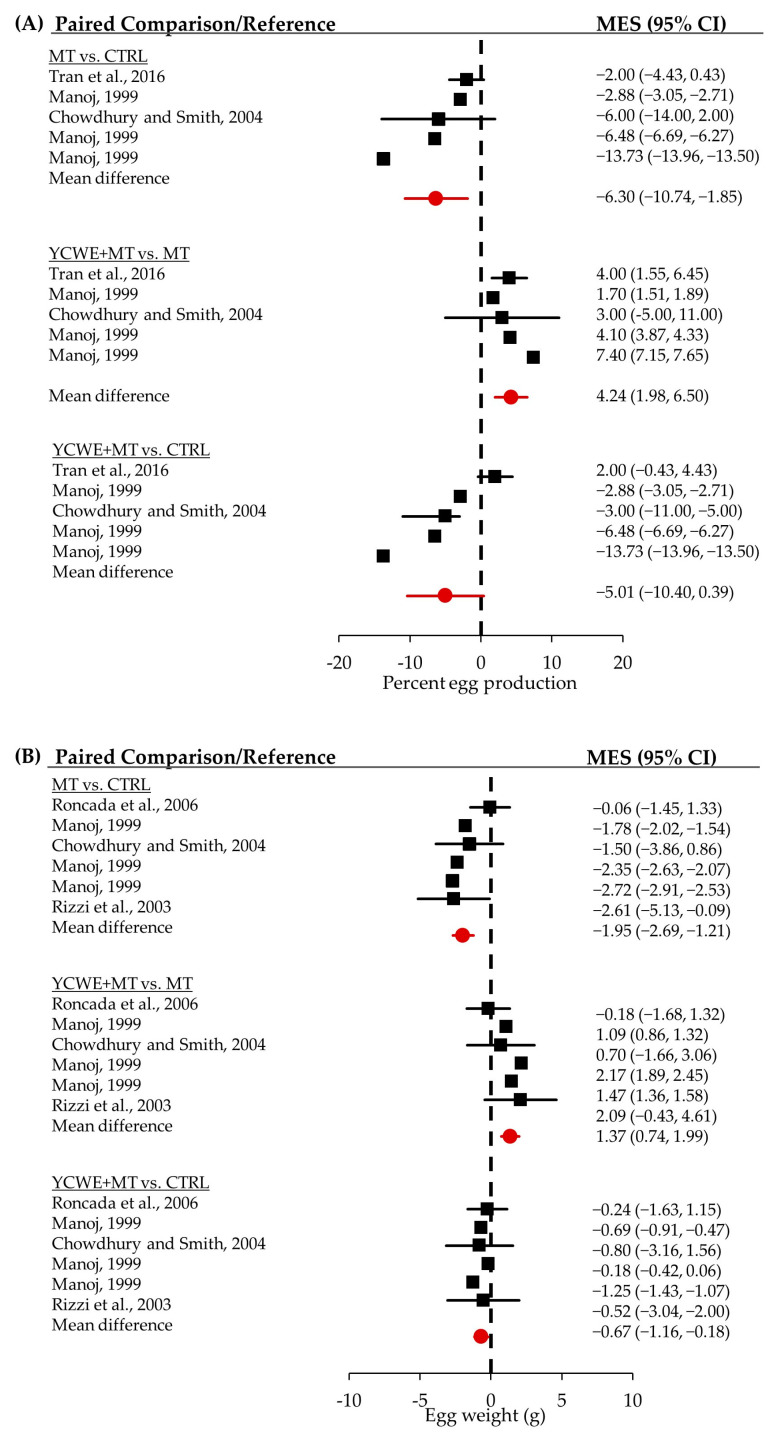
Forest plots from the random-effects meta-analysis [18,19,20,21,22,22,23] of the mean differences in (**A**) percent egg production, and (**B**) egg weight for laying hens fed control without detectable mycotoxins (CTRL), mycotoxins alone (MT), or yeast cell wall extract during mycotoxin challenges (YCWE + MT, Mycosorb^®^, Alltech, Inc.). The mean effect sizes (MES) with 95% confidence intervals (95% CI) between treatments are shown for individual studies (in black) and the overall difference (in red).

Hens fed MT had significantly reduced (*p* < 0.01) egg production than those fed CTRL by −6.3 percentage points (Table 2, Figure 2). However, the feeding of YCWE + MT resulted in significantly greater (*p* < 0.001) egg production by a mean difference of 4.2 percentage points compared with hens fed MT. The egg production rate of hens fed YCWE + MT was closer to CTRL but did still have a tendency (*p* = 0.069) to be lower. 

### 2.5. Egg Weight 

Egg weight was decreased significantly (*p* < 0.0001) by a mean difference of −1.95 g for birds consuming MT versus CTRL (Table 2, Figure 2). Hens fed YCWE + MT had significantly greater (*p* < 0.0001) egg weight by 1.37 g than those fed MT. The egg weight from hens fed YCWE + MT remained lower than CTRL eggs by −0.67 g (*p* < 0.05). 

### 2.6. Between-Study Heterogeneity and Publication Bias

Publication bias and heterogeneity (*I*^2^) were assessed in this meta-analysis (Table 2). Forest plots are provided in Supplementary Materials Figures S1–S3. Publication bias was investigated by the Eggar’s test for asymmetry which showed no significance (*p* > 0.05) for BW, egg production, or egg weight. For FI, there was indication of potential publication bias (*p* < 0.05) for MT versus CTRL and YCWE + MT versus MT. Between-study *I*^2^ indicated significant differences (*p* < 0.0001) for all treatment comparisons for BW, FI, and egg production, with *I*^2^ values above 99%. The *I*^2^ values were between 87.9% to 94.3% for egg weight. Wide ranges of *I*^2^ are reported in other published meta-analyses [26,27,28,29]. One meta-analysis was able to further explore unaccounted *I*^2^ through meta-regression, which investigated the effect of mycotoxin concentration on broiler performance response [29]. Due to a limited number of studies in the current meta-analysis, as well as most trials investigating only higher levels of mycotoxins, further breakdown of data by category was not possible.

### 2.7. Economic Assessment

Economic assessment was calculated based on the mean effects size obtained from the meta-analysis and using a time frame of 9.5 weeks, the average number of weeks across all trials. Calculations (Table 3) showed that hens fed MT had lower performance output than hens fed CTRL or YCWE + MT. When comparing the difference between YCWE + MT and MT, the inclusion of YCWE over an average of 9.5 weeks resulted in 2.7 more eggs per hen housed, cumulatively, 240 g greater egg mass total, 6.90 g lower feed per egg produced, and a calculated 29.69 g rise in edible protein output per hen. Additionally, there was a positive calculated return on investment (ROI) of 4.65:1.

## *3.* Discussion

The frequent presence of mycotoxins in feed commodities globally has been well described [5,6]. The consumption of these mycotoxins by poultry may harm health and performance with potential impacts that include alteration of gut structure and microbial functions, suppression of immunity and response to vaccinations, reduced body weight gain, induction of oxidative stress, or lower egg production and quality [30,31,32,33,34]. These negative effects to laying hens may occur even when mycotoxin concentrations are at levels below EU-proposed limits, as indicated by Kulcsar et al. [32]. Although mycotoxin challenges are present on-farm, management strategies such as the use of feed additives can be employed to mitigate the harmful effects of mycotoxins on poultry [28,35]. Yeast cell wall extract contains insoluble carbohydrates associated with a complex parietal network that have demonstrated affinity for the physico-chemical interaction with mycotoxins [36,37]. Adsorptive properties for mycotoxins primarily occur from the β-D-glucan network present in the yeast cell wall which can generate multiple van der Waals and hydrogen bonds with mycotoxins. Research has shown YCWE to have adsorptive properties for numerous mycotoxins including but not limited to AFB1, DON, T2, FUM, ZEA, emerging mycotoxins, and *Penicillium* mycotoxins [11,14,38]. Recently, two meta-analyses have been published demonstrating the use of YCWE during mycotoxin challenges for broilers and growing pigs [28,29]. Using data from 8 trials involving laying hens, the novel meta-analysis presented herein was further able to support the use of YCWE during mycotoxin challenges.

Based on the studies included in this meta-analysis, consumption of MT diets lowered hen BW by a mean difference of −49.98 g compared to CTRL birds. Subsequently, egg weight was also reduced for birds consuming mycotoxins by a mean of −1.95 g and a 95% confidence interval range of −2.69 g to −1.21 g. The loss in egg weight could be expected, as one of the factors influencing egg weight is hen body weight [39]. Changes to egg weight may influence farm economics. If egg weight fluctuates, a portion of the eggs produced may drop a size class thus resulting in reduced profitability [40,41]. Based on the results of this meta-analysis, a lowering of egg weight by up to almost 3 g could be enough to result in a class change, particularly for those eggs already on the outer ranges of a weight class. 

Profitability may also be influenced by egg production. This meta-analysis showed that hens consuming mycotoxin-contaminated feed had a decrease in egg production by a mean difference of −6.3 compared with CTRL. Egg production has been classified as the single most important indicator of the performance of laying hens [39]. As such, the impact of mycotoxins on reducing production could have an impact on the entire farm’s productivity and profitability. Although bird response to mycotoxins can be variable, numerous trials have shown an influence on production performance [33,42]. Additionally, the presence of multiple mycotoxins together may further impact production and health. For example, Jia et al. [43] showed that laying hens consuming AF alone had lowered production rates while ZEA alone tended to lower production, but the combination of these two mycotoxins together had an even greater impact on reducing laying performance. Similarly, hens consuming feed naturally contaminated with aflatoxin B1 (AFB1), DON, FUM, T2, and ZEA had lower egg production by −4.8 to −14.9% [44], while feeding a combination of AFB1, DON, and OTA reduced egg production by −5.3% [45]. Although the mean difference for egg production determined in the current meta-analysis falls within these ranges, these trials investigating multi-mycotoxin challenges indicate that production loss could be greater than what is suggested by the meta-analysis. Furthermore, it should be noted that not only can egg production rates be lowered by mycotoxin presence, but so can egg quality as mycotoxins have been shown to alter the fatty acid composition and antioxidant status of egg yolks [46]. As such, eggs that are produced may not pass quality controls or produce healthy chicks. 

A number of different feed additives are available that may be used for mitigation of mycotoxins. These products can include inorganic compounds such as bentonites and aluminosilicate clays, yeast material including yeast cell wall, enzymes, and bacteria [47]. The focus of the current meta-analysis was on the use of the yeast product, YCWE, which was previously reported to adsorb a wide variety of mycotoxins, mycotoxin mixtures, and provide benefits to broilers such as supporting performance, lowering mortality, and an increasing European Poultry Efficiency Factor during mycotoxin challenges [28,47]. Studies with laying hens and mycotoxins are limited compared with other species, with research involving mycotoxin management strategies for layers being even more limited. It is clear however, that there is an investment in research for understanding how YCWE plays a role in supporting layer performance during mycotoxin challenges as the literature search for this meta-analysis resulted in finding 21 unique trials in this area (8 utilized here for meeting selection requirements). As such, a meta-analysis of these trials can be helpful in gaining a more practical overview for the use of YCWE during mycotoxin challenge situations in laying hens. 

Results from this meta-analysis indicated that the inclusion of YCWE during mycotoxin challenges had the greatest impact on both egg production and egg weight. Whereas mycotoxins lowered the laying rate, the use of YCWE resulted in significant production benefits and recovered 67.3% of the lost egg production during the mycotoxin challenges (increase in mean difference of 4.2 percentage points versus MT). That could equate to about 2.7 extra eggs/hen over a 9.5-week laying period. These data represent the overall mean of the trials used in this meta-analysis; however, the mycotoxin exposure and challenge duration did vary between trials. As such, the production response could be less than or greater than the mean reported. 

Furthermore, along with greater egg production, there was also higher egg weight for hens fed YCWE + MT. Calculations for the impact of this effect over 9.5 weeks resulted in an estimated 240 g more egg mass per hen housed. As a result of lowered egg production and egg weight by MT, the total edible protein output of production could also be decreased. Based on calculations from this meta-analysis, the average total edible protein output decreased by 43.93 g per hen housed over the average 9.5-week period. Although the true reduction in total edible protein could be more variable than the decrease reported herein, these data do suggest that mycotoxins may play a role in protein output. Eggs can be an important component of a healthy and sustainable diet. They are typically inexpensive, provide a source of high-quality protein, vitamins, and minerals, and may be regarded as a solution to undernutrition in many parts of the world [48]. Furthermore, eggs are reported to have the lowest global warming potential per average daily intake compared to other major animal-derived products (beef > pork > chicken > milk > eggs) [49]. Egg production is also estimated to have the lowest associated land and energy use as compared to other animal proteins. Although already one of the more sustainable industries, egg production continues to become more efficient through enhanced resource efficiencies, improved animal health, and changes to welfare such as production shifts to cage-free [50,51]. As such, maintaining egg production when unsuspected commodities containing mycotoxins are fed to hens is of importance to both human health and environmental sustainability. To combat a loss in production, this meta-analysis showed that YCWE inclusion in the ration may help alleviate mycotoxin challenge through greater egg production, egg weight, and thereby higher total edible protein output by an estimated average of 29.69 g per hen housed over a 9.5-week period. While this is the calculated estimate, individual variations in egg production or egg weight between trials or within trials, could influence the final effect on-farm. 

Due to the production performance benefits that occurred when including YCWE in the ration at an average rate of 1.58 kg/ton, the calculated ROI was 4.65:1. This is a valuable return given the range of mycotoxin types and concentrations that the challenged hens were subjected to in their rations, with maximum mycotoxin levels of up to 1 mg/kg AF [21], 12.1 mg/kg DON [18], 2.0 mg/kg T2 [19], 25 mg/kg FUM [21], and 10.4 mg/kg ZEA [22]. In the future, research that investigates the effect of mycotoxins with YCWE on laying hens at more common commercial levels of mycotoxin challenge will be beneficial. Furthermore, the calculated effects on profitability and ROI in this study were based solely on egg production parameters. However, YCWE use during mycotoxin challenge may provide benefits to other aspects of layer production such as egg quality and antioxidant status [20,46], immunity [52], organ health [18,19], or livability, which also affect total output and economic returns. As such, additional research with laying hens investigating not only YCWE use but also simply the effect of lower levels of mycotoxins, could be insightful for producers whose flocks may experience lower concentrations of mycotoxins on a daily basis.

## 4. Conclusions

Feedstuffs used in the poultry industry are likely to be contaminated with some level of mycotoxins. The results reported in the present meta-analysis show that consumption of these mycotoxins can have negative effects on laying hen body weight, egg production rate, and egg weight. With a wide range of mycotoxin types and contamination levels from the trials used herein, dietary inclusion of YCWE resulted in greater egg production and egg weights. Additionally, economic assessment supports the use of YCWE through higher hen output and positive ROI. As such, the inclusion of YCWE in diets of hens exposed to mycotoxin challenges could be recommended to support performance and profitability.

## 5. Materials and Methods

### 5.1. Literature Search and Selection

Following a similar outline to previously published research by Weaver et al. [28], a literature search was undertaken to collate all available published research and unpublished trial reports that evaluated the use of YCWE (Mycosorb^®^, Alltech Inc., Nicholasville, KY, USA) inclusion during mycotoxin challenges on the performance of laying hens. Although numerous mitigation agents are available to the poultry industry, this meta-analysis focused on assessing the use of YCWE as a single management solution. Yeast cell wall extract is produced from *Saccharomyces cerevisiae*, with the major components being β-(1,3)-D-glucans and β-(1,6)-D-glucans which represent between 25% to 30% of the yeast cell wall biomass and have shown to have adsorption properties for mycotoxins [29]. Additional components of the yeast cell wall include mannoproteins and chitin. These cell wall components form a covalently linked three-dimensional network surrounding the entire yeast cell [36,37]. Clay material within this product does not exceed 2% and is present as an anticaking agent. 

**Figure 3:**
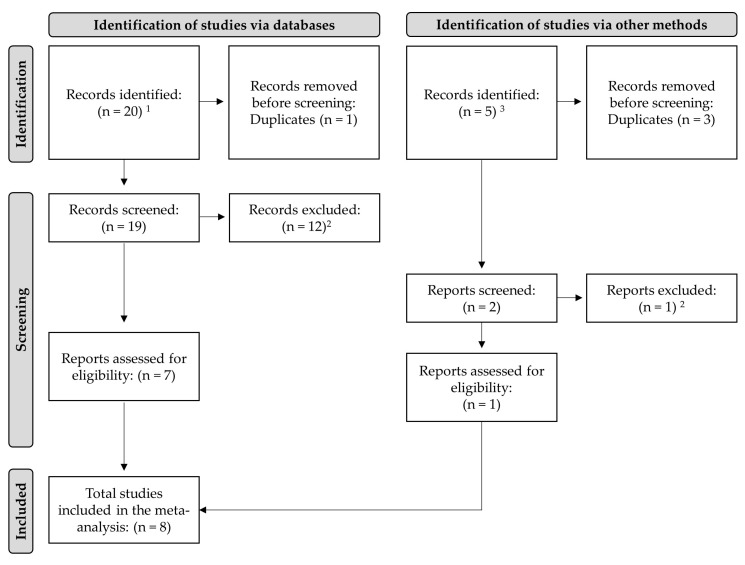
A PRISMA flow diagram showing the identification, screening, and final inclusion of trials for this random-effects meta-analysis investigating the impacts of mycotoxins with or without supplementation of yeast cell wall extract (Mycosorb^®^, Alltech Inc.) on the performance of laying hens. ^1^ Search of references was conducted in electronic databases using specific inclusion criteria. ^2^ Reasons for exclusion were: (1) lack of mycotoxin results reported, or (2) performance parameters not measured or reported. ^3^ Organizational data obtained from Alltech’s internal database.

A literature search was conducted in January 2023 from online databases (Google Scholar, Agricola, PubMed) using keywords of “mycotoxins”, “Alltech”, and “layers”, “breeders”, “hens”, “yeast cell wall”, “esterified glucomannan”, “glucomannan polymer”, or “Mycosorb^®^.” Unpublished trial reports were obtained from Alltech’s internal bibliography database. Unpublished reports were included to reduce the potential for publication bias as described by Duval and Tweedie [53]. There were no date restrictions and no restrictions on mycotoxin type or concentration. Following the literature search, research was subject to screening by the following criteria: (1) trials must be conducted with laying hens or broiler breeder chickens; (2) there must contain at least one mycotoxin challenge treatment as well as at least one treatment with the specific YCWE product being investigated used during a mycotoxin challenge; (3) the inclusion of CTRL treatment without added mycotoxins, undetectable mycotoxins, or mycotoxin content significantly below treatment challenges was desired but not required; (4) at least one mycotoxin concentration needed to be provided, as well as the YCWE inclusion rate; (5) at least one variable of performance including body weight (BW), feed intake (FI), egg production percentage, or egg weight; and (6) the number of days in the experimental period and the number of birds in the trial was reported. Results from each trial were partitioned into treatments of either CTRL, MT, or YCWE + MT. This systemic review process is shown in a PRISMA flow diagram as described by Page et al. [54] (Figure 3).

### 5.2. Statistical Analysis

A random-effects meta-analysis was conducted using R [55], R Studio [56], and the package *metafor* (META-analysis FOr R) [57] following methods previously described by Weaver et al. [28]. A meta-analysis is a statistical model providing a quantitative method to assess and integrate the results of individual research trials into one overall conclusion [58]. The meta-analysis model can be shown as:

(1) $${\hat{\theta }}_{i}=\mu +e_{i}+\zeta_{i}\;$$

with the variable $\hat{\theta }$_i_ representing the observed effect size in the *i*-th study, *μ* representing an average of the true effect size from a distribution of effect sizes, *e_i_* being the sampling error with *e_i_* ~ *N* (0, *v*_i_), and ζ*_i_* as the error associated with the distribution of effect sizes, or the between-study heterogeneity, with **ζ***_i_* ~ *N* (0, τ^2^). Standard deviations (SD) reported in each study were used to calculate the sampling variance (*v*_i_). In instances where the coefficient of variation (CV) was reported rather than SD, the SD was determined by multiplying the mean by the CV. If neither the CV nor SD were reported, an SD was computed as the average SD from the other studies for each treatment group. Variance that was associated with effect size (**τ**^2^) distribution was estimated using restricted maximum likelihood [59]. There was no transformation of the data to assume normally distributed estimates.

Publication bias was assessed through the generation of funnel plots (Supplement Figures S1–S3) and further quantified through the use of Eggar tests. Funnel plots are depicted as scatter plots that graph the individual trial effect estimates which are plotted against study precision [26]. The Egger test examines asymmetry in funnel plots and may be used to quantify publication bias [60]. The between-study heterogeneity (*I*^2^) was evaluated, which is a measure of variability between studies [61,62,63]. Heterogeneity may be detected if variation is greater than what is expected by random chance. This statistic is calculated as:

(2) $$I^{2}=\frac{Q-df}{Q}\times 100\%$$

where variable *Q* is the calculated chi-squared statistic and *df* is the degrees of freedom associated with a comparison. The *I*^2^ variable can be used to describe unaccounted differences in mean effect sizes between the treatments (CTRL, MT, and YCWE + MT) for each of the assessed response variables (BW, FI, egg production, egg weight). Heterogeneity is lower with smaller (<50%) values of *I*^2^, while higher *I*^2^ is indicated by larger (>50%) values [58,61]. Alpha values less than 0.05 were considered significant and between 0.05 and 0.10 as tendencies.

### 5.3. Economic Assessment

Potential impact on economic return was conducted based on the average value of feed and eggs [64] for conventional layer production in the United States during 2023. Calculations were assessed per hen housed based on data obtained from this meta-analysis, including results for BW, FI, egg production, and egg weight. A 9.5-week laying period was used, which was the average number of weeks for trials used in this meta-analysis. Using these calculations, the ROI was calculated for the use of YCWE during mycotoxin challenges. The ROI was calculated as the net profit increase per hen housed divided by the product cost per hen housed based on a product inclusion rate of 1.58 kg/ton which was the average inclusion rate used by trials included in this meta-analysis. Additionally, the influence of mycotoxins or YCWE on the total edible protein output was calculated based on the number of eggs produced per hen and the average total protein content per egg which was derived from the mean egg weight reported in this meta-analysis and a whole egg protein content of 12.6% [65,66].

## Figures and Tables

**Table 1:** Description of studies utilized for the random-effects meta-analysis examining the impact of mycotoxins without or with yeast cell wall extract on the performance of laying hens.

									Mycotoxin, mg/kg ^6^							
Ref. ^1^	Location	Breed	Hens/trt ^2^	Start Age, Weeks	Trial Weeks	Number of MT Levels ^3^	YCWE, kg/t ^4^	Source ^5^	AF, AFB1	OTA	DON	15-A-DON	DON-3-G	T2	FUM, FB1	ZEA
[18]	Canada	ISA Brown	48	45	12	1	2	Natural			12.1	0.5				0.60
[19]	India	-	24	26	12	1	1	Pure						0.5		
						2	1							1.0		
						3	1							2.0		
[20]	Canada	Lohmann LS-LITE	36	48	12	1	2	Natural			2.53		0.31			0.33
[21]	Brazil	Hisex Brown	24	37	8	1	2	Culture	1.0							
						2	2								25	
						3	2		1.0						25	
[22]	Italy	Warren	18	-	4	1	1.1	-	0.893						0.171	10.36
[23]	Italy	Warren-ISA Brown	14	-	12	1	2	Pure		0.20						
[24] ^7^	Serbia	Shaver 579	500	18	12	1	2	Natural	0.005	0.19						3.14
[25]	USA	Cobb	30	35	4	1	0.91	Culture	3.0							

^1^ Ref.: reference number, information not supplied indicated by “-”. ^2^ Hens/trt: number of birds per treatment from control, mycotoxin, or mycotoxin plus yeast cell wall (Mycosorb^®^, Alltech, Inc.). ^3^ Number of MT levels: different mycotoxin concentrations within a trial. ^4^ YCWE, kg/t: inclusion of yeast cell wall extract in kg per ton. ^5^ Source: contamination from naturally contaminated feedstuffs, artificial culture, or pure mycotoxins. ^6^ AF: aflatoxins; AFB1: aflatoxin B1; OTA: ochratoxin A; DON: deoxynivalenol; 15-A-DON: 15-acetyl-deoxynivalenol; DON-3-G: deoxynivalenol-3-glucoside; T2: T-2 toxin; FUM: fumonisins; FB1: fumonisin B1; ZEA: zearalenone. ^7^ Feeding trial began with 1-day-old chicks, egg production measured from 18 weeks of age, average mycotoxin level over the entire experimental period.

**Table 2:** Mean effect size estimates for the random-effects meta-analysis examining the impacts of mycotoxins without or with yeast cell wall extract on the performance of laying hens.

					Heterogeneity Test		
Item ^1^	No. Comp. ^2^	Mean Effect Size	95% CI ^3^	*p*-Value	*I*^2^ (%) ^4^	*p*-Value	Eggar *p*-Value ^5^
Body weight, g							
MT − CTRL	6	−49.98	−88.77, −11.20	0.012	99.96	<0.0001	0.871
YCWE + MT − MT	7	12.47	−19.25, 44.19	0.441	99.97	<0.0001	0.121
YCWE + MT − CTRL	6	−25.57	−52.60, −2.54	0.031	99.89	<0.0001	0.202
Feed intake, g/d							
MT − CTRL	8	−1.21	−8.06, 5.65	0.730	99.78	<0.0001	0.003
YCWE + MT − MT	8	−0.80	−4.41, 2.82	0.666	99.07	<0.0001	0.009
YCWE + MT − CTRL	8	−2.47	−5.91, 0.96	0.158	99.26	<0.0001	0.064
Egg production, %							
MT − CTRL	6	−6.30	−10.74, −1.85	0.006	99.91	<0.0001	0.732
YCWE + MT − MT	6	4.24	1.98, 6.50	<0.001	99.52	<0.0001	0.779
YCWE + MT − CTRL	6	−5.01	−10.40, 0.39	0.069	99.94	<0.0001	0.482
Egg weight, g							
MT − CTRL	6	−1.95	−2.69, −1.21	<0.001	94.30	<0.0001	0.292
YCWE + MT − MT	6	1.37	0.74, 1.99	<0.001	93.59	<0.0001	0.406
YCWE + MT − CTRL	6	−0.67	−1.16, −0.18	0.008	87.86	<0.0001	0.745

^1^ CTRL: control diets without added mycotoxins or undetectable mycotoxin contamination; MT: diets with reported mycotoxin challenge; and YCWE + MT: diets containing both yeast cell wall extract (Mycosorb^®^, Alltech, Inc.) and mycotoxins. Mean effects size estimates for treatment differences were determined between MT minus CTRL, YCWE + MT minus MT, and YCWE + MT minus CTRL. ^2^ No. Comp.: number of different trial comparisons available for each treatment and performance variable. ^3^ 95% CI: 95% confidence interval; ^4^ *I*^2^: between-study variation. ^5^ Eggar *p*-Value: Eggar test for asymmetry to assess publication bias.

**Table 3:** Economic analysis per hen housed based on the mean effect size results obtained from the random-effects meta-analysis examining the impact of mycotoxins without or with yeast cell wall extract inclusion.

	Treatments ^1^			
Item	CTRL	MT	YCWE + MT	YCWE + MT vs. MT ^2^
Number of weeks laying	9.5	9.5	9.5	-
Cumulative egg counts per hen	62.51	58.52	61.18	2.66
Egg mass per hen, kg	3.72	3.37	3.60	0.24
Edible protein per egg, g	7.49	7.25	7.42	0.17
Edible protein output, g	468.20	424.27	453.96	29.69
Cumulative feed intake per hen, kg	7.59	7.53	7.45	−0.08
Grams of feed per egg, g	121.42	128.67	121.77	−6.90
Average egg weight, g	59.48	57.54	58.91	1.37
Egg price per egg produced, USD	0.125	0.125	0.125	-
Total value of eggs sold per hen, USD	7.81	7.32	7.65	0.33
Feed cost per ton, USD	316.09	316.09	323.99	7.90
Product inclusion rate, kg	-	-	1.58	-
Feed cost per hen, USD	2.40	2.38	2.41	0.03
Return on investment (ROI)				4.65:1

^1^ CTRL: control diets without added mycotoxins or undetectable mycotoxin contamination; MT: diets with reported mycotoxin challenge; and YCWE + MT: diets containing both yeast cell wall extract (Mycosorb^®^, Alltech, Inc.) and mycotoxins. ^2^ Difference calculated for YCWE + MT minus MT.

## Data Availability

The original contributions presented in the study are included in the article/Supplementary Material, further inquiries can be directed to the corresponding author.

## Supplementary Materials

The following supporting information can be downloaded at: https://www.mdpi.com/article/10.3390/toxins16040171/s1, Figure S1: Funnel plots for assessment of the publication bias of studies included in the random-effects meta-analysis showing the mean differences in (**A**) body weight (g), (**B**) feed intake (g/d), (**C**) percent egg production, and (**D**) egg weight (g) of laying hens. Circles represent individual study comparisons between treatments of control (CTRL) versus mycotoxins alone (MT).; Figure S2: Funnel plots for assessment of the publication bias of studies included in the random-effects meta-analysis showing the mean differences in (**A**) body weight (g), (**B**) feed intake (g/d), (**C**) percent egg production, and (**D**) egg weight (g) of laying hens. Circles represent individual study comparisons between treatments of yeast cell wall extract during mycotoxin challenges (YCWE + MT, Mycosorb^®^, Alltech, Inc.) versus mycotoxins alone (MT).; Figure S3: Funnel plots for assessment of the publication bias of studies included in the random-effects meta-analysis showing the mean differences in (**A**) body weight (g), (**B**) feed intake (g/d), (**C**) percent egg production, and (**D**) egg weight (g) of laying hens. Circles represent individual study comparisons between treatments of control (CTRL) versus yeast cell wall extract during mycotoxin challenges (YCWE + MT, Mycosorb^®^, Alltech, Inc.).
