# Supplementary material for: Meta-Analysis of the Effects of Yeast Cell Wall Extract Supple-Mentation during Mycotoxin Challenges on the Performance of Laying Hens
Source: Toxins (Basel). 2024 Mar 30;16(4):171. doi: 10.3390/toxins16040171 (PMC11054775; doi:10.3390/toxins16040171)
Supplement: Supplementary file 1 [file toxins-16-00171-s001.zip › toxins-2918329-supplementary.pdf]

Meta-analysis of the effects of yeast cell wall extract supple-mentation during mycotoxin challenges on the performance of laying hens

Alexandra C. Weaver, Daniel M. Weaver, Nicholas Adams and Alexandros Yiannikouris

SUPPLEMENTARY MATERIAL

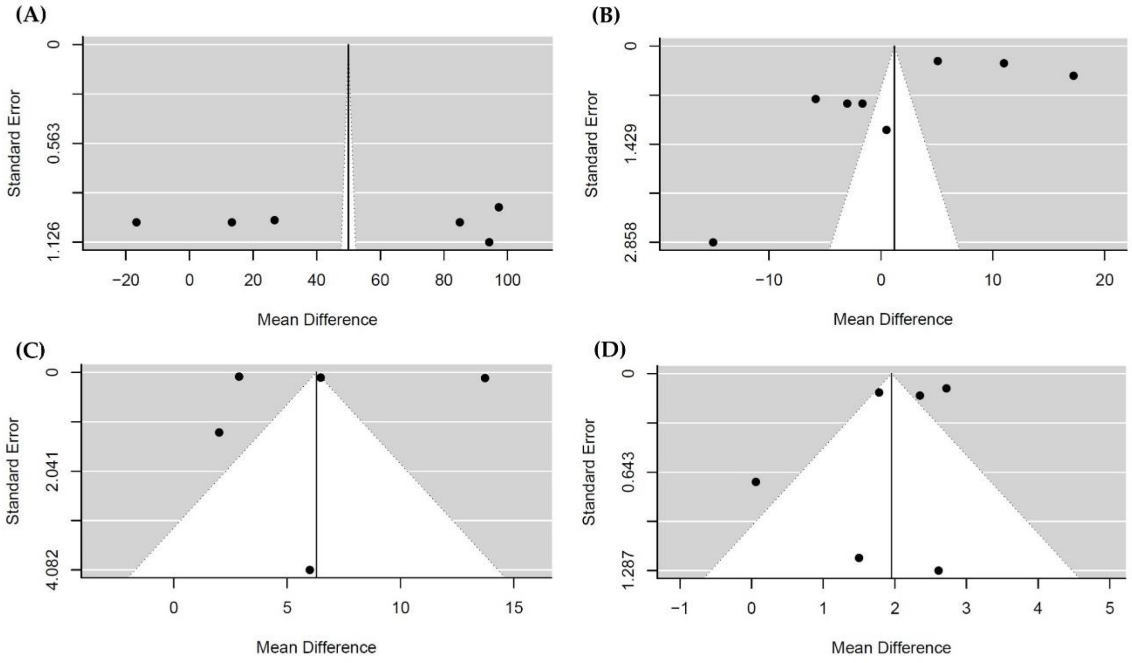

Supplementary Figure S1. Funnel plots for assessment of the publication bias of studies included in the random-effects meta-analysis showing the mean differences in (**A**) body weight (g), (**B**) feed intake (g/d), (**C**) percent egg production, and (**D**) egg weight (g) of laying hens. Circles represent individual study comparisons between treatments of control (CTRL) versus mycotoxins alone (MT).

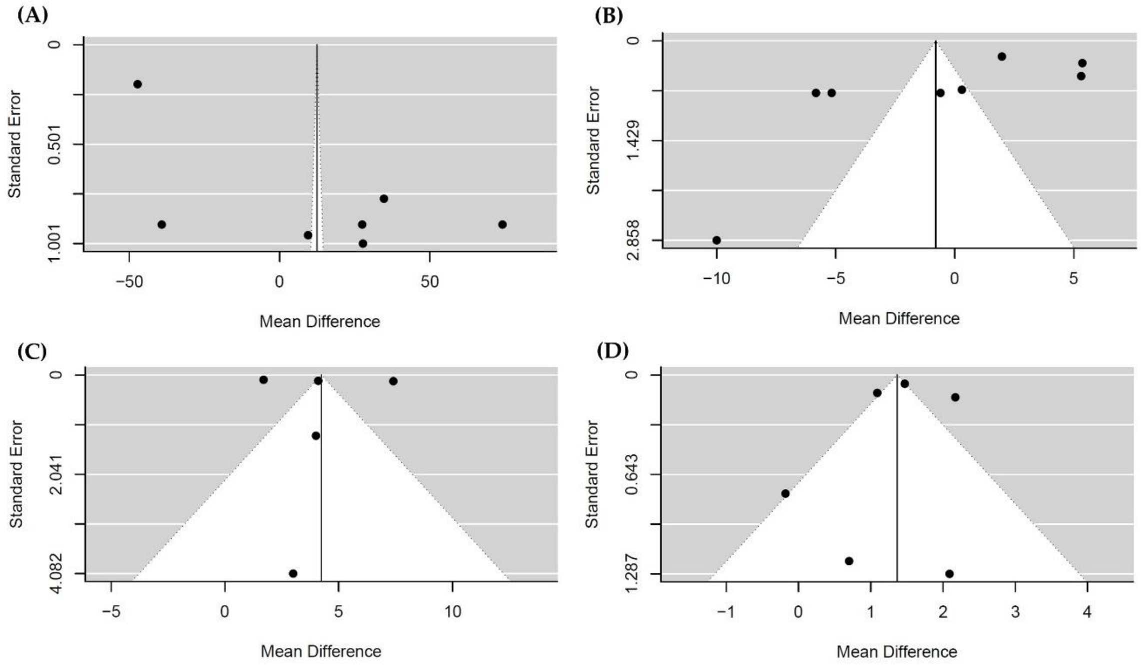

Supplementary Figure S2. Funnel plots for assessment of the publication bias of studies included in the random-effects meta-analysis showing the mean differences in (**A**) body weight (g), (**B**) feed intake (g/d), (**C**) percent egg production, and (**D**) egg weight (g) of laying hens. Circles represent individual study comparisons between treatments of yeast cell wall extract during mycotoxin challenges (YCWE+MT, Mycosorb®, Alltech, Inc.) versus mycotoxins alone (MT).

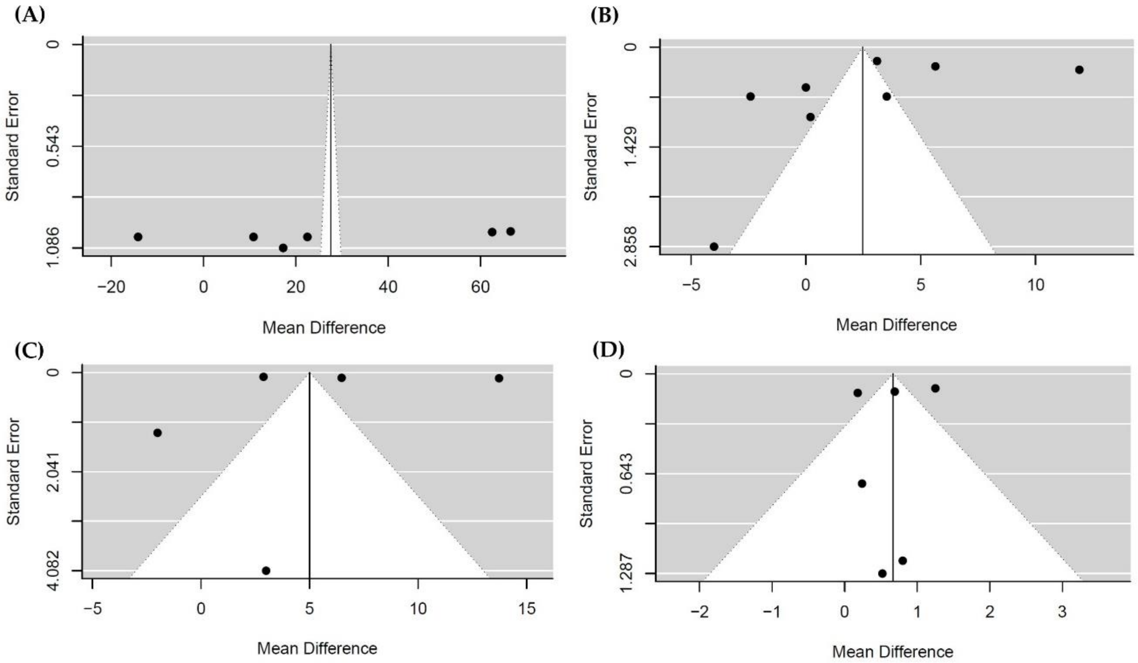

Supplementary Figure S3. Funnel plots for assessment of the publication bias of studies included in the random-effects meta-analysis showing the mean differences in (**A**) body weight (g), (**B**) feed intake (g/d), (**C**) percent egg production, and (**D**) egg weight (g) of laying hens. Circles represent individual study comparisons between treatments of control (CTRL) versus yeast cell wall extract during mycotoxin challenges (YCWE+MT, Mycosorb®, Alltech, Inc.).
